# Somatic Mutation–Informed Transcriptional Architecture of Hepatocellular Carcinoma: A Multiomics Study Centered on *TP53*

**DOI:** 10.1155/humu/2377057

**Published:** 2026-10-09

**Authors:** Sheng Zhang, Guojun Zhang, Wenxuan Wang, Hong Luo, Huaping Chen

**Affiliations:** ^1^ Department of Radiology Beijing Anzhen Nanchong Hospital of Medical University and Nanchong Central Hospital Nanchong China; ^2^ Department of Oncology Beijing Anzhen Nanchong Hospital of Medical University and Nanchong Central Hospital Nanchong China

**Keywords:** hepatocellular carcinoma, multiomics integration, single-cell RNA sequencing, somatic mutation, *TP53* mutation, transcriptional heterogeneity, tumor genomics

## Abstract

Hepatocellular carcinoma (HCC) is characterized by extensive somatic mutation heterogeneity, yet the cellular contexts in which recurrent driver mutation–associated transcriptional programs operate remain incompletely defined. In this study, we applied a mutation‐informed multiomics framework centered on *TP53* to connect somatic mutation status, bulk transcriptional programs, and hepatocyte‐like cell states at single‐cell resolution. Somatic mutation profiles and transcriptomic data from the TCGA‐LIHC cohort were used to construct directional mutation‐associated transcriptional signatures, with *CTNNB1* retained as a reference driver signature and *AXIN1* and *ARID1A* retained for descriptive comparison. These bulk‐derived transcriptional programs were then projected into single‐cell RNA sequencing data to evaluate inferred *TP53*‐associated transcriptional activity across hepatocyte‐like populations. *TP53* mutation‐associated transcriptional programs were enriched in proliferation‐related pathways, DNA repair processes, and stress–response programs and were associated with adverse clinical outcomes. At the single‐cell level, inferred *TP53*‐associated transcriptional activity showed heterogeneous distribution across hepatocyte‐like populations and was preferentially enriched in putative malignant hepatocyte‐like states. Pseudobulk and functional program analyses further supported the association between *TP53*‐high signature states, cell‐cycle activity, DNA repair, stress programs, and altered tumor‐cell communication patterns. Integrated prioritization identified *CDCA8*, *MCM10*, and *TRIP13* as representative genes associated with *TP53*‐related transcriptional programs; these genes should be interpreted as components of a *TP53*‐associated proliferative transcriptional state rather than as experimentally validated drivers. These findings provide a mutation‐informed view of transcriptional architecture in HCC and illustrate how bulk mutation‐associated signatures can be integrated with single‐cell data to improve biological interpretation of somatic variation.

## 1. Introduction

Hepatocellular carcinoma (HCC) is characterized by substantial genomic complexity and remains a leading cause of cancer‐related mortality worldwide [1, 2]. Large‐scale sequencing studies have demonstrated that HCC harbors recurrent somatic mutations affecting key regulatory pathways involved in cell cycle control, Wnt signaling, chromatin remodeling, and genome stability [3, 4]. Among these, *TP53* and *CTNNB1* represent the most frequently altered driver genes and define major molecular subclasses of HCC [5, 6]. Although the genomic landscape of HCC has been extensively described, the extent to which recurrent somatic mutations shape tumor transcriptional organization and cellular heterogeneity remains incompletely understood [7].

*TP53* functions as a central regulator of genomic stability through its roles in DNA damage response, apoptosis, and cell cycle regulation [8]. *TP53* mutation is consistently associated with aggressive tumor phenotypes, increased proliferative activity, and poor clinical outcomes in HCC [9, 10]. Previous studies have identified transcriptional differences between *TP53*‐mutant and wild‐type tumors; however, these findings have largely been derived from bulk tumor analyses [11, 12]. Because bulk transcriptomic profiles represent averaged signals across heterogeneous tumor cell populations, the cellular contexts in which mutation‐associated transcriptional programs operate remain insufficiently characterized [13].

Recent advances in multiomics approaches provide new opportunities to connect genomic alterations with transcriptional and cellular phenotypes [14]. Integration of somatic mutation data with bulk transcriptomics enables systematic characterization of mutation‐associated transcriptional programs, whereas single‐cell RNA sequencing allows the resolution of tumor cell heterogeneity at cellular resolution [15, 16]. These complementary approaches enable investigation of how mutation‐associated transcriptional programs are distributed across tumor cell populations. However, integrative analyses that connect mutation‐derived transcriptional signatures with hepatocyte transcriptional states at the single‐cell level remain limited.

In this study, we characterized the transcriptional architecture associated with recurrent somatic mutations in HCC through a multiomics analytical framework centered on *TP53*. Compared with prior bulk analyses of *TP53*‐associated expression differences, this study emphasizes the placement of mutation‐associated transcriptional programs within hepatocyte‐like single‐cell states. By deriving directional mutation‐associated signatures from TCGA‐LIHC and projecting them into an independent single‐cell atlas, we aim to localize *TP53*‐associated transcriptional activity within the cellular heterogeneity of HCC. This mutation‐informed strategy provides a framework for interpreting downstream transcriptional consequences of recurrent somatic mutations and for prioritizing candidate genes supported by convergent bulk, single‐cell, and clinical evidence.

## 2. Materials and Methods

### 2.1. Study Design and Data Sources

The overall objective of this study was to link recurrent driver mutations in HCC to downstream transcriptional consequences and hepatocyte‐like transcriptional states identified at single‐cell resolution. The workflow was designed to relate recurrent mutations to transcriptional programs and cellular states rather than to establish causal oncogenic drivers. Somatic mutation profiles, RNA sequencing data, and clinical annotations were obtained from the TCGA‐LIHC cohort [17]. Mutation annotation format (MAF) files and RNA‐seq STAR count data were downloaded from the Genomic Data Commons portal [18]. Clinical annotations and survival information were harmonized using UCSC Xena resources when necessary to ensure consistency of overall survival definitions [19]. Single‐cell RNA sequencing data from GSE149614 were used to characterize hepatocyte transcriptional states across normal liver tissue, primary tumors, portal vein tumor thrombus (PVTT), and lymph node samples [13]. The GSE14520 dataset was additionally used as an external bulk‐level source for candidate‐gene expression and clinical support when available [20]. All data analyzed in this study were obtained from publicly accessible repositories with prior ethical approval.

### 2.2. Somatic Mutation Analysis and Candidate Driver Selection

Somatic mutation data from TCGA‐LIHC were processed at the primary‐tumor patient level. Only variants predicted to have functional impact were retained, including missense mutations, nonsense mutations, frameshift insertions or deletions, splice‐site variants, in‐frame insertions or deletions, translation initiation variants, and nonstop mutations, whereas synonymous mutations were excluded. Mutation frequencies were calculated as the proportion of primary‐tumor patients harboring at least one retained nonsynonymous or functional variant in each gene. Therefore, the reported *TP53* and *CTNNB1* frequencies may differ slightly from those in the original TCGA‐LIHC publication because of patient‐level counting, restriction to primary tumors, and exclusion of synonymous variants. Mutation summaries, including mutation frequency distributions, variant classification, single‐nucleotide variant spectra, tumor mutation burden distributions, and pairwise mutation interaction analyses, were generated using maftools [21]. Tumor mutation burden was calculated as the number of nonsynonymous mutations per megabase based on an estimated 38‐Mb coding territory and was log‐transformed and standardized before inclusion as a covariate in downstream analyses [22]. *TP53* was selected as the central mutation for downstream single‐cell projection because of its high prevalence, well‐established clinical relevance in HCC, association with adverse outcomes, and strong bulk transcriptional signal in the present analysis. *CTNNB1* was retained as a reference driver signature because it represents a biologically distinct major HCC subclass, whereas *AXIN1* and *ARID1A* were retained for descriptive comparison but not used for downstream signature construction due to lower mutation frequencies and limited statistical power.

### 2.3. Mutation‐Associated Differential Expression Modeling

RNA sequencing counts were restricted to protein‐coding genes and filtered to remove low‐expression genes using edgeR filterByExpr. Library size normalization was performed using the trimmed mean of M‐values method [23], followed by limma‐voom modeling [24, 25]. For each candidate mutation, differential expression was modeled using a design matrix of the form expression ~ mutation status + sex + pathologic stage + standardized log1p tumor mutation burden, with terms omitted only when a covariate had insufficient variation in the analyzed subset. The mutation coefficient therefore represented the adjusted expression difference between mutant and wild‐type tumors. To account for repeated RNA‐seq files from the same patient when present, duplicateCorrelation was applied with patient identifier as the blocking variable [26]. Empirical Bayes moderation was performed with trend and robust options. Genes with false discovery rate below 0.05 and absolute log2 fold change exceeding log2(1.30) were considered mutation‐associated transcriptional changes. The log2(1.30) threshold was selected as a moderate effect‐size filter intended to retain biologically interpretable transcriptional differences while avoiding signatures dominated by genes with very small effects. To prioritize genes with consistent and biologically meaningful differences, genes were ranked using a composite score defined as absolute log2 fold change multiplied by ‐log10(adjusted *p* value).

### 2.4. Construction of Directional Mutation Transcriptional Signatures

For each mutation, the top 40 genes upregulated in mutant tumors and the top 40 genes downregulated in mutant tumors were selected from significant mutation‐associated genes to construct directional transcriptional signatures. Genes were ranked within each direction by the composite score described above. The balanced 40‐up and 40‐down design was chosen to represent coordinated bidirectional transcriptional shifts while keeping the signature compact enough for projection across bulk and single‐cell datasets. Directional signature scores were calculated as follows. For a sample or cell *i*, expression of each matched signature gene *g* was standardized across all analyzed samples or cells to obtain z_gi. The up‐score was the mean z_gi across matched upregulated genes, the down‐score was the mean z_gi across matched downregulated genes, and the combined directional score was defined as up‐score minus down‐score. Signature genes absent from a given expression matrix were excluded from scoring; genes with zero variance after standardization were assigned a *z*‐score of zero. Scores were subsequently standardized for downstream regression analyses when appropriate.

### 2.5. Bulk‐Level Validation of Mutation Signatures

At the patient level, duplicate RNA‐seq samples were collapsed by averaging log2 counts‐per‐million values to avoid sample‐level redundancy. Mutation classification ability of directional signature scores was evaluated using Wilcoxon tests, receiver operating characteristic analysis, and logistic regression. Adjusted logistic regression models included signature score, sex, tumor stage, and standardized log1p tumor mutation burden when data availability permitted. Overall survival analyses were conducted using Kaplan–Meier methods with median score dichotomization. Cox proportional hazards models were constructed both with and without adjustment for age, sex, tumor stage, and tumor mutation burden. To evaluate biological context, Hallmark gene‐set activity was quantified by averaging gene‐wise standardized expression values for matched genes in each Hallmark program [27], and associations between signature scores and program scores were evaluated using Spearman correlation. This analysis should be distinguished from the ranked gene‐set enrichment analysis used in the single‐cell pseudobulk comparison. Immune and stromal transcriptional programs were quantified using curated marker‐based gene sets for cytolytic activity, CD8 T cells, regulatory T cells, NK cells, B cells, dendritic cells, macrophages, fibroblast/CAF programs, endothelial cells, extracellular matrix remodeling, interferon‐gamma response, and antigen presentation.

### 2.6. Single‐Cell RNA Sequencing Analysis and Hepatocyte State Definition

Single‐cell RNA sequencing data from GSE149614 were processed using Seurat [28]. Genes detected in fewer than three cells were excluded during count‐matrix construction. Cells were retained if they had at least 300 detected genes, at least 500 UMIs, a mitochondrial transcript percentage no greater than 20%, and nFeature_RNA and nCount_RNA values below the 99th percentile of the full dataset. Doublet removal and ambient RNA correction were not performed because the analysis used publicly available processed count matrices and author‐provided metadata; this limitation was considered when interpreting cell‐state assignments. After log‐normalization with a scale factor of 10,000, highly variable genes were identified using the vst method. The full atlas was analyzed using 3000 variable genes, regression of nCount_RNA and mitochondrial percentage during scaling, 50 principal components, Dimensions 1–30 for graph construction and UMAP, and clustering resolution 0.40. Cell type annotations provided by the original study were used as the starting point for downstream analysis and were checked using canonical marker genes.

The hepatocyte compartment was extracted and independently reclustered to better resolve tumor‐associated transcriptional states. The hepatocyte subset was reprocessed using log‐normalization, 2500 variable genes, regression of nCount_RNA and mitochondrial percentage, 40 principal components, Dimensions 1–20, and clustering resolution 0.35. Because copy number inference was not incorporated into the final workflow, malignant hepatocyte‐like states were defined operationally rather than as directly validated malignant clones. Specifically, hepatocyte clusters were assigned as normal‐like, putative malignant hepatocyte‐like, or intermediate based on tissue origin, the fraction of cells from tumor‐related sites, reduced normal liver transcriptional programs, and increased oncofetal, proliferative, or stress/hypoxia programs. Normal hepatocytes were retained as reference populations.

### 2.7. Projection of Mutation Signatures Into Single‐Cell Data

Bulk‐derived *TP53* and *CTNNB1* transcriptional signatures were projected into the hepatocyte compartment using the same directional scoring framework. For each cell, log‐normalized expression values of matched signature genes were standardized across cells before calculation of directional scores. These scores were interpreted as inferred *TP53*‐ or *CTNNB1*‐associated transcriptional activity rather than as direct single‐cell mutation calls, because mutation status was not measured at single‐cell resolution in GSE149614. Within putative malignant hepatocyte‐like cells, *TP53*‐high and *TP53*‐low populations were defined based on the upper and lower quartiles of *TP53* signature scores, with intermediate cells defined as those between these thresholds. Module scores for proliferation, cell cycle activity, stress response, oncofetal programs, and normal liver programs were calculated to evaluate transcriptional associations and potential nonspecific proliferative or technical effects.

### 2.8. Pseudobulk Differential Expression Analysis

To reduce donor‐specific bias inherent to single‐cell data, pseudobulk profiles were generated by aggregating counts from *TP53* signature‐high and *TP53* signature‐low putative malignant hepatocyte‐like cells within each patient [29]. Only patients contributing at least 15 cells to both high‐ and low‐score pseudobulk groups were retained to ensure balanced comparisons. Differential expression analysis was performed using edgeR and limma‐voom with a paired design of the form expression ~ patient + *TP53* score group [30]. Genes meeting false discovery rate and fold‐change thresholds were considered differentially expressed. Functional interpretation included comparison of curated module scores and Hallmark gene‐set enrichment analysis using fgsea with Hallmark gene sets from msigdbr, ranked by the moderated t‐statistic from the pseudobulk comparison.

### 2.9. Cell–Cell Communication Analysis

Cell–cell communication analysis was performed using CellChat to computationally infer potential interactions between putative malignant hepatocyte‐like cells and other tumor‐associated cell populations [31]. Communication groups included *TP53* signature‐high malignant hepatocyte‐like cells, *TP53* signature‐low malignant hepatocyte‐like cells, other malignant hepatocyte‐like cells, nonmalignant hepatocytes, immune populations, endothelial cells, and fibroblasts. Groups with insufficient cell numbers were excluded, and remaining groups were downsampled to reduce bias from unequal group sizes. Communication probability was inferred using the human CellChat database. These analyses were treated as exploratory computational predictions of ligand–receptor communication rather than experimentally validated cell–cell interactions.

### 2.10. Candidate Gene Prioritization

Candidate genes were prioritized from genes upregulated in *TP53* signature‐high putative malignant hepatocyte‐like cells using a stepwise evidence integration strategy. Genes were required to be detectable in single‐cell data, enriched in *TP53*‐high cells, positively correlated with the *TP53* signature score, supported by TCGA bulk expression differences between *TP53*‐mutant and wild‐type tumors, correlated with bulk *TP53* signature scores, and associated with poorer prognosis when survival data were available. To reduce prioritization bias toward generic proliferation genes, canonical broad cell‐cycle genes were removed using a predefined blacklist, and additional penalties were applied to genes with strong correlations with proliferation, cell‐cycle, or stress scores. CellChat support and GSE14520 external bulk evidence were incorporated as supportive but not definitive evidence. The final candidates were therefore interpreted as computationally prioritized components of *TP53*‐associated transcriptional programs rather than experimentally confirmed functional drivers.

### 2.11. Statistical Analysis

All analyses were performed in R Version 4.5.1. Statistical tests were two‐sided unless otherwise specified. Multiple testing correction was performed using the Benjamini–Hochberg procedure. Group comparisons were performed using Wilcoxon or Kruskal–Wallis tests where appropriate; for the stage comparisons in Figure 1B, a Kruskal–Wallis test was performed separately for each signature, and the resulting *p* values were adjusted across the two signatures using the Benjamini–Hochberg method. Associations between continuous variables were evaluated using Spearman correlation. Survival analyses were performed using Kaplan–Meier estimation and Cox proportional hazards modeling. Key nondefault analytical parameters are described in the corresponding methods sections; otherwise, package default settings were used. Key packages included data.table 1.18.2.1, dplyr 1.2.0, ggplot2 4.0.2, edgeR 4.6.3, limma 3.64.3, Seurat 5.4.0, CellChat 1.6.1, fgsea 1.34.2, msigdbr 25.1.1, survival 3.8.6, survminer 0.5.1, maftools 2.24.0, ComplexHeatmap 2.24.1, and circlize 0.4.17.

**Figure 1:**
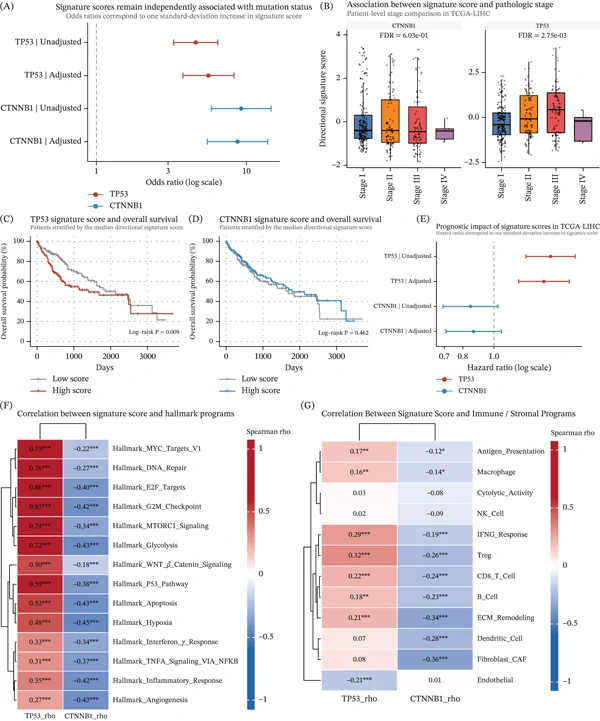
Clinical and biological associations of mutation‐associated transcriptional signatures. (A) Logistic regression analysis showing associations between mutation status and directional signature scores before and after adjustment for clinical covariates. (B) Association between directional signature scores and tumor pathologic stage. (C) Kaplan–Meier analysis of overall survival stratified by *TP53* signature scores. (D) Kaplan–Meier analysis of overall survival stratified by *CTNNB1* signature scores. (E) Cox regression analysis showing hazard ratios associated with signature scores in unadjusted and adjusted models. (F) Correlation between signature scores and Hallmark pathway activities. (G) Correlation between signature scores and immune and stromal transcriptional programs.

## 3. Results

### 3.1. Somatic Mutation Landscape and Candidate Driver Gene Characterization in HCC

To define the genomic background for mutation‐associated transcriptional analyses, we first characterized the somatic mutation landscape of HCC in the TCGA‐LIHC cohort using primary‐tumor patient‐level nonsynonymous and functional variants. Among 368 primary tumors, 312 samples (84.78%) harbored at least one nonsynonymous mutation in the analyzed gene set, indicating substantial interpatient genomic heterogeneity (Figure 2A). Mutation frequency analysis showed that *TP53* (28.5%) and *CTNNB1* (25.5%) were the most frequently mutated genes, followed by *TTN* (24.2%), *MUC16* (16.0%), *ALB* (12.8%), and *PCLO* (11.1%) (Figure 2B). These frequencies reflect the filtering strategy used in this study and are therefore not expected to be identical to unfiltered frequencies reported in earlier TCGA summaries. Among established HCC driver genes, *AXIN1* and *ARID1A* exhibited mutation frequencies of 7.1% and 7.3%, respectively. Based on mutation prevalence, biological relevance, and downstream statistical power, *TP53* was selected as the central mutation for integrated analysis, and *CTNNB1* was retained as a reference driver signature, whereas *AXIN1* and *ARID1A* were retained as additional recurrent drivers for descriptive comparison (Figure 2C,D). These four genes were altered in 215 of 368 tumors (58.42%). Variant classification analysis showed that missense mutations represented the dominant mutation category, followed by frameshift deletions, nonsense mutations, and frameshift insertions (Figure 2E). At the variant type level, single nucleotide variants constituted the majority of detected alterations, with smaller contributions from insertions and deletions. Analysis of substitution classes showed that C > T transitions represented the most frequent substitution pattern. Tumor mutation burden showed a right‐skewed distribution, with most tumors exhibiting relatively low mutation counts and a smaller subset showing elevated mutation levels (Figure 2F). Substitution pattern analysis further showed a predominance of transition mutations relative to transversions (Figure 2G). Pairwise interaction analysis showed mutually exclusive patterns between *TP53* and *CTNNB1* mutations. *AXIN1* mutations also showed mutual exclusivity with *CTNNB1*, whereas *ARID1A* mutations showed limited interaction patterns with the other candidate drivers (Figure 2H).

**Figure 2:**
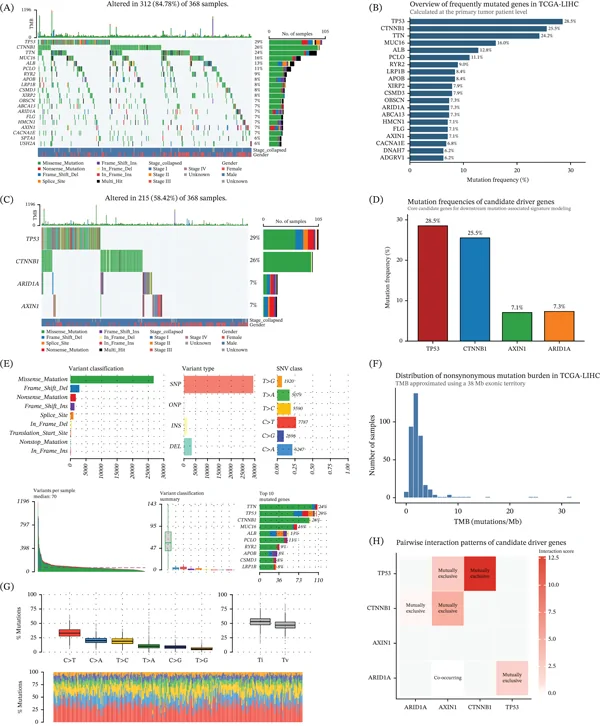
Somatic mutation landscape and characterization of recurrent driver genes in hepatocellular carcinoma. (A) Oncoplot showing the mutation landscape of frequently mutated genes in TCGA‐LIHC. Each column represents one tumor sample, and each row represents one gene. Mutation types are indicated by color. Clinical annotations including tumor stage and gender are shown at the bottom. (B) Mutation frequencies of the most frequently mutated genes calculated at the primary tumor level. (C) Mutation patterns of selected candidate driver genes (*TP53*, *CTNNB1*, *AXIN1*, and *ARID1A*). (D) Mutation frequencies of candidate driver genes used for downstream mutation‐associated analyses. (E) Summary of variant classifications, variant types, single nucleotide variant classes, mutation counts per sample, and top mutated genes. (F) Distribution of nonsynonymous tumor mutation burden in TCGA‐LIHC samples. (G) Distribution of single nucleotide substitution classes and transition/transversion patterns. (H) Pairwise interaction analysis of candidate driver genes showing mutually exclusive and co‐occurring mutation patterns.

### 3.2. Mutation‐Associated Transcriptional Signatures Stratify Mutation Status in HCC

To characterize transcriptional programs associated with major driver mutations, directional expression signatures were constructed for *TP53* and *CTNNB1* based on adjusted mutation‐associated differential expression analysis. For each mutation, significant genes were ranked by a composite of effect size and statistical significance, and balanced signatures consisting of up to 40 upregulated and 40 downregulated genes were retained. Directional signature scores were significantly higher in mutation carriers compared with wild‐type tumors for both *TP53* and *CTNNB1*, indicating that the derived gene sets captured mutation‐associated transcriptional differences (Figure 3A). Receiver operating characteristic analysis further showed that these signatures distinguished mutation status with AUC values of 0.803 for *TP53* and 0.915 for *CTNNB1* (Figure 3B). Differential expression analysis identified mutation‐associated transcriptional differences for both genes. For *TP53*, a subset of genes showed increased expression in mutant tumors, whereas another subset showed reduced expression (Figure 3C). A similar bidirectional expression pattern was observed for *CTNNB1* mutation‐associated genes (Figure 3E). Heatmap visualization showed that mutation‐associated genes separated tumors into transcriptional groups consistent with mutation status for both *TP53* and *CTNNB1* (Figure 3D–F). Cross‐comparison of *TP53* and *CTNNB1* signature scores showed that *TP53*‐mutant tumors tended to exhibit higher *TP53* signature scores and lower *CTNNB1* signature scores, whereas *CTNNB1*‐mutant tumors showed the opposite pattern (Figure 3G). Based on the predefined balanced selection criteria, 80 genes were retained for each mutation‐associated signature, consisting of 40 genes upregulated and 40 genes downregulated in mutant tumors (Figure 3H).

**Figure 3:**
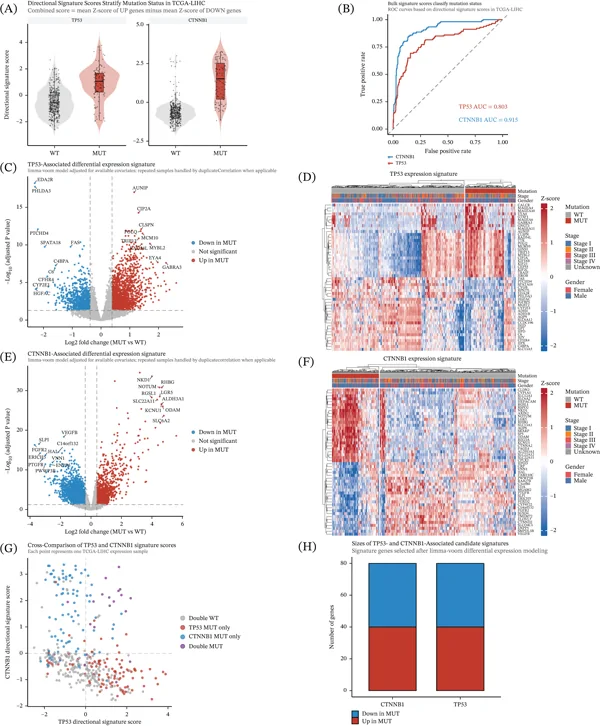
Construction and validation of mutation‐associated transcriptional signatures. (A) Directional signature scores comparing mutant and wild‐type tumors for *TP53* and *CTNNB1*. (B) Receiver operating characteristic curves evaluating classification performance of mutation‐associated signatures. (C) Volcano plot showing differential gene expression associated with *TP53* mutation. (D) Heatmap showing expression patterns of *TP53*‐associated signature genes across TCGA‐LIHC samples. (E) Volcano plot showing differential gene expression associated with *CTNNB1* mutation. (F) Heatmap showing expression patterns of *CTNNB1*‐associated signature genes across TCGA‐LIHC samples. (G) Cross‐comparison of *TP53* and *CTNNB1* directional signature scores across samples. (H) Numbers of genes included in *TP53* and *CTNNB1* mutation‐associated signatures.

### 3.3. Clinical and Biological Associations of Mutation‐Associated Transcriptional Signatures

Logistic regression analysis showed that both *TP53* and *CTNNB1* signature scores remained significantly associated with mutation status after adjustment for available clinical covariates (Figure 1A). The adjusted models showed similar effect sizes compared with unadjusted models, indicating that the associations were not driven by clinical confounders. Association analysis with pathologic stage showed significant differences in signature scores across tumor stages for both *TP53* and *CTNNB1* (Figure 1B). *TP53* signature scores tended to be higher in more advanced tumors, whereas *CTNNB1* signature scores showed an inverse distribution pattern across stages. Survival analysis showed that tumors with higher *TP53* signature scores had significantly worse overall survival compared with those with lower scores (log‐rank *p* = 0.009) (Figure 1C). In contrast, *CTNNB1* signature scores were not significantly associated with overall survival (Figure 1D). Cox regression analysis showed that *TP53* signature scores were associated with increased hazard of death in both unadjusted and adjusted models, whereas *CTNNB1* signature scores showed no significant survival association (Figure 1E). Correlation analysis with Hallmark gene sets showed that *TP53* signature scores were positively correlated with multiple proliferation‐associated programs, including MYC targets, DNA repair, E2F targets, and G2M checkpoint signatures (Figure 1F). *CTNNB1* signature scores showed negative correlations with several of these programs. Correlation analysis with immune and stromal transcriptional programs showed that *TP53* signature scores were positively associated with several immune‐related programs, whereas *CTNNB1* signature scores showed negative correlations with multiple immune and stromal programs (Figure 1G).

### 3.4. Single‐Cell Characterization of Hepatocyte Transcriptional States in HCC

To characterize hepatocyte transcriptional states associated with mutation‐derived programs, single‐cell RNA sequencing data from GSE149614 were analyzed. Quality control assessment showed comparable distributions of detected genes, transcript counts, and mitochondrial gene proportions across tissue sites (Figure 4A). Cell clustering identified major cell populations including hepatocytes, T/NK cells, myeloid cells, B cells, endothelial cells, and fibroblasts (Figure 4B). Tissue‐site mapping showed the distribution of cells from normal liver, tumor, PVTT, and lymph node samples within the single‐cell atlas (Figure 4C). Cell‐type annotations were supported by expression of canonical marker genes, including *ALB* and *APOA1* for hepatocytes, *CD3D* for T cells, *MS4A1* for B cells, *PECAM1* for endothelial cells, and *COL1A1* for fibroblasts (Figure 4E). Cell composition analysis showed substantial variation across tissue sites. Hepatocytes represented the dominant population in tumor and PVTT samples, whereas immune populations were more prominent in normal liver and lymph node samples (Figure 4D). To further resolve hepatocyte heterogeneity, hepatocytes were subsetted and reclustered, identifying multiple hepatocyte subclusters (Figure 4F). These clusters showed distinct tissue distributions, with some clusters enriched in tumor samples and others predominantly observed in normal liver tissue (Figure 4G,I). Based on transcriptional program patterns, hepatocyte clusters were grouped into normal‐like hepatocytes, putative malignant hepatocyte‐like populations, and intermediate states (Figure 4H). Program‐level analysis showed differences across clusters in liver functional programs, stress‐related programs, oncofetal programs, and proliferation‐associated transcriptional programs (Figure 4J). Expression patterns of representative marker genes further supported these state assignments, with proliferation markers such as *MKI67* enriched in specific clusters, whereas liver functional genes such as *ALB* and *APOA1* were enriched in other clusters (Figure 4K).

**Figure 4:**
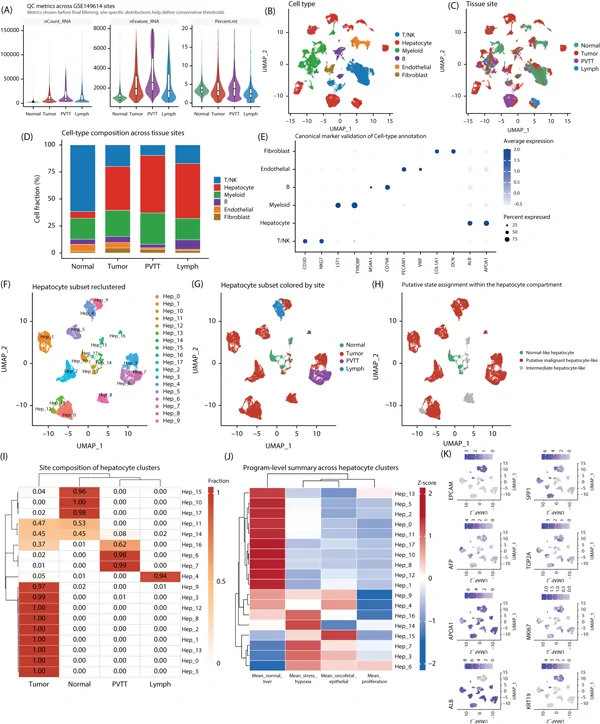
Single‐cell characterization of hepatocyte transcriptional states in hepatocellular carcinoma. (A) Quality control metrics across tissue sites in GSE149614. (B) UMAP visualization showing cell‐type annotations. (C) UMAP visualization showing tissue origin. (D) Cell‐type composition across tissue sites. (E) Canonical marker validation of cell‐type annotation. (F) Reclustering of hepatocyte populations showing hepatocyte subclusters. (G) Distribution of hepatocyte clusters across tissue sites. (H) Putative state assignment within the hepatocyte compartment. (I) Site composition of hepatocyte clusters. (J) Program‐level summary across hepatocyte clusters. (K) Expression of representative marker genes across hepatocyte clusters.

### 3.5. Projection of Mutation‐Associated Transcriptional Signatures Into Hepatocyte Single‐Cell States

To evaluate whether mutation‐associated transcriptional programs could be localized within hepatocyte‐like single‐cell states, bulk‐derived *TP53* signature scores were projected onto the hepatocyte compartment. These scores represent inferred *TP53*‐associated transcriptional activity rather than direct mutation status at the single‐cell level. *TP53* signature activity showed heterogeneous distribution across hepatocyte clusters and was enriched in clusters classified as putative malignant hepatocyte‐like populations (Figure 5A). Within putative malignant hepatocyte‐like cells, *TP53* and *CTNNB1* signature scores showed variable distributions across cells (Figure 5B). Comparison of *TP53* and *CTNNB1* directional signature scores showed a weak inverse relationship across hepatocytes (Spearman rho = −0.05) (Figure 5C). Within the putative malignant hepatocyte‐like population, *TP53* signature scores showed heterogeneous distribution across clusters. High‐score cells defined by the upper quartile of *TP53* signature scores were primarily localized to specific hepatocyte clusters (Figure 5D). Cluster‐level analysis showed variation in *TP53*‐associated transcriptional programs across hepatocyte clusters (Figure 5E). Correlation analysis showed that *TP53* signature scores were positively associated with generic transcriptional programs including cell cycle, proliferation, and stress‐related programs (Figure 5F). Comparison between *TP53* high‐ and low‐score hepatocytes showed differences in multiple transcriptional programs. High‐score cells showed higher activity of proliferation and stress‐related programs, whereas low‐score cells showed higher activity of liver functional programs (Figure 5G). These observations support localization of *TP53*‐associated transcriptional activity but do not establish single‐cell *TP53* mutation status.

**Figure 5:**
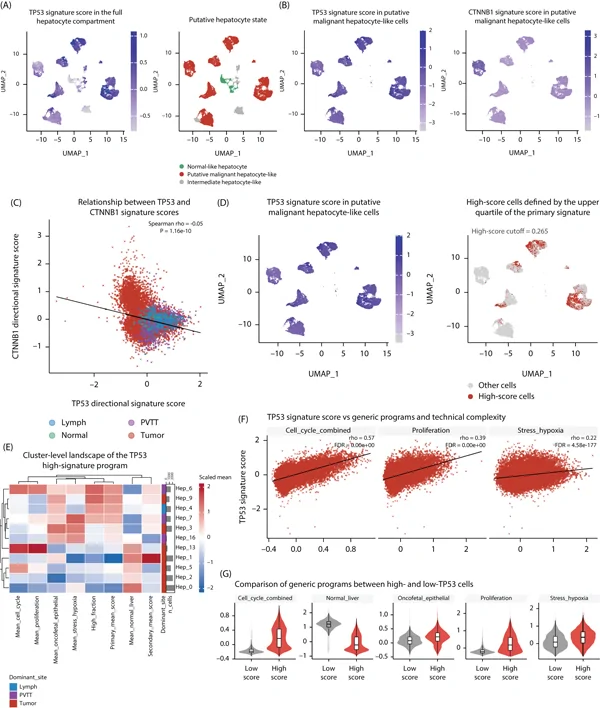
Projection of mutation‐associated transcriptional signatures into hepatocyte single‐cell states. (A) *TP53* signature score distribution across the hepatocyte compartment and putative hepatocyte state assignments. (B) Distribution of *TP53* and *CTNNB1* signature scores within putative malignant hepatocyte‐like populations. (C) Relationship between *TP53* and *CTNNB1* signature scores across hepatocytes. (D) Distribution of *TP53* signature scores within putative malignant hepatocyte‐like hepatocytes and identification of high‐score cells. (E) Cluster‐level landscape of *TP53* high‐signature programs across hepatocyte clusters. (F) Correlation between *TP53* signature scores and generic transcriptional programs. (G) Comparison of generic transcriptional programs between *TP53* high‐score and low‐score hepatocytes.

### 3.6. Pseudobulk and Cell Interaction Analyses of *TP53* High‐Signature Hepatocyte Populations

To reduce donor‐specific bias, pseudobulk profiles were generated by aggregating putative malignant hepatocyte‐like cells according to *TP53* signature score groups within each patient. Principal component analysis showed separation between *TP53* high‐score and low‐score groups across patients (Figure S1A). Differential expression analysis identified genes upregulated and downregulated between *TP53* high‐score and low‐score malignant hepatocyte‐like cells (Figure S1B). Heatmap visualization of top differentially expressed genes showed consistent expression differences across pseudobulk samples (Figure S1C). Representative genes showed distinct expression patterns between *TP53* high‐ and low‐score groups (Figure S1D). Comparison of transcriptional programs showed that *TP53* high‐score malignant hepatocyte‐like cells exhibited higher activity of EMT, stemness, hypoxia, and glycolysis‐related programs, whereas oxidative phosphorylation and normal liver programs showed higher activity in low‐score cells (Figure S1E). Gene set enrichment analysis further showed enrichment of proliferation‐related programs including E2F targets, G2M checkpoint, MYC targets, and DNA repair in *TP53* high‐score cells, whereas metabolic programs were enriched in *TP53* low‐score cells (Figure S1F). These results indicate that the *TP53*‐high state is strongly linked to proliferative and cell‐cycle transcriptional activity, and this relationship was considered when prioritizing candidate genes. Correlation analysis showed coordinated relationships between *TP53* signature scores and multiple functional transcriptional programs, including glycolysis, hypoxia, DNA repair, and proliferation programs (Figure S1G). Patient‐level comparisons showed consistent differences between *TP53* high‐score and low‐score cells across multiple functional programs (Figure S1H). To evaluate potential differences in cell interaction patterns, exploratory CellChat analysis was performed across tumor‐associated cell populations. *TP53* high‐signature malignant hepatocyte‐like cells showed distinct predicted communication patterns relative to *TP53* low‐signature malignant hepatocyte‐like cells (Figure S1I, S1J). Analysis of signaling pathways suggested differences in predicted communication probabilities involving *TP53* high‐score malignant cells (Figure S1K). Ligand–receptor analysis identified multiple predicted interactions involving *TP53* high‐score malignant hepatocyte‐like cells and other cell populations (Figure S1L). Aggregate communication analysis showed differences in incoming and outgoing communication strength between *TP53* high‐score and *TP53* low‐score malignant hepatocyte‐like cells (Figure S1M). These communication results should be interpreted as computational predictions.

### 3.7. Prioritization and Computational Support for *TP53*‐Associated Candidate Genes

To identify candidate genes associated with *TP53*‐related transcriptional programs, a stepwise prioritization framework was applied. Starting from 701 *TP53*‐upregulated genes, filtering based on detection in the single‐cell dataset, cell‐type specificity, TCGA bulk support, confounder filtering, and prognostic association progressively reduced the candidate list to three final genes (Figure 6A). The confounder filtering step removed predefined broad cell‐cycle genes and penalized genes with strong correlations with proliferation, cell‐cycle, or stress programs, thereby reducing prioritization of genes driven only by generic mitotic activity. Integrated ranking based on multicriteria evaluation identified *CDCA8*, *MCM10*, and *TRIP13* as the top candidate genes among *TP53*‐associated genes (Figure 6B). These genes showed consistently higher expression in *TP53* high‐score malignant hepatocyte‐like cells compared with low‐score cells (Figure 6C). Single‐cell visualization further showed that *CDCA8*, *MCM10*, and *TRIP13* were preferentially expressed in subsets of hepatocyte clusters corresponding to putative malignant hepatocyte‐like populations (Figure 6D). Bulk TCGA analysis showed higher expression of these genes in *TP53*‐mutant tumors compared with *TP53* wild‐type tumors (Figure 6E). Expression levels of these genes were strongly positively correlated with *TP53* signature scores (Figure 6F). Correlation analysis showed associations between expression of the final candidate genes and multiple transcriptional programs, including DNA repair, E2F targets, and G2M checkpoint programs (Figure 6G). Survival analysis showed that higher expression of *CDCA8*, *MCM10*, and *TRIP13* was associated with poorer overall survival (Figure 6H). Time‐dependent ROC analysis showed moderate prognostic performance for these genes across follow‐up time points (Figure 6I). Because these genes are closely linked to cell‐cycle biology, they are best interpreted as representative markers of a *TP53*‐associated proliferative transcriptional program pending experimental validation.

**Figure 6:**
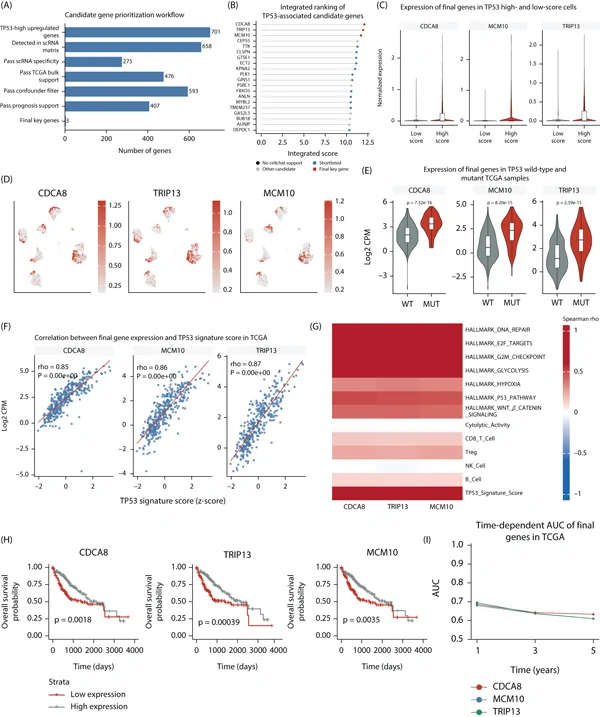
Prioritization and computational support for *TP53*‐associated candidate genes. (A) Candidate gene prioritization workflow showing sequential filtering steps. (B) Integrated ranking of *TP53*‐associated candidate genes. (C) Expression of final candidate genes in *TP53* high‐ and low‐score malignant hepatocytes. (D) Single‐cell expression distribution of *CDCA8*, *TRIP13*, and *MCM10*. (E) Expression of final candidate genes in *TP53* wild‐type and mutant TCGA samples. (F) Correlation between candidate gene expression and *TP53* signature scores. (G) Correlation between candidate gene expression and transcriptional programs. (H) Kaplan–Meier survival analysis of final candidate genes. (I) Time‐dependent ROC analysis of final candidate genes.

## 4. Discussion

Understanding how recurrent somatic mutations shape tumor transcriptional organization remains an important challenge in cancer genomics [32]. Although previous studies have characterized *TP53*‐associated expression differences in HCC, most have relied primarily on bulk tumor profiles. The present study advances this literature by connecting mutation‐associated bulk transcriptional signatures with hepatocyte‐like single‐cell states and candidate‐gene prioritization across multiple computational evidence layers. Thus, the main novelty of the study lies in the mutation‐informed integration across genomic, bulk transcriptomic, and single‐cell transcriptional scales rather than in the identification of *TP53*‐associated differential expression alone.

Our results show that *TP53* mutation is associated with coordinated transcriptional programs related to proliferation, DNA damage response, and cellular stress pathways. These findings are consistent with the established biological role of *TP53* in maintaining genomic stability and regulating cell cycle progression [33, 34]. Loss of *TP53* function has been shown to promote genomic instability and uncontrolled cell proliferation, which may explain the enrichment of proliferation‐associated transcriptional programs observed in *TP53*‐associated transcriptional signatures. In addition, the observed associations with DNA repair and stress–response pathways further support the central role of *TP53* in coordinating cellular responses to genomic damage [34].

By projecting mutation‐associated transcriptional programs into single‐cell data, our study further suggests that *TP53*‐associated transcriptional activity is not uniformly distributed across hepatocyte‐like populations but is preferentially enriched in putative malignant hepatocyte‐like states characterized by increased proliferative and stress‐related transcriptional activity [35]. Because mutation status was inferred from bulk‐derived transcriptional programs rather than directly measured in individual cells, these results should be interpreted as localization of *TP53*‐associated transcriptional activity rather than direct identification of *TP53*‐mutant single cells. This distinction is important because bulk transcriptomic analyses may average signals across heterogeneous cell populations, whereas single‐cell projection provides a cellular context for interpreting mutation‐associated transcriptional programs [36].

The integrative multiomics framework applied in this study also provides a strategy for linking genomic alterations with transcriptional organization across biological scales [37]. By combining mutation status, transcriptional programs, and cellular context, this approach allows systematic characterization of how recurrent genomic alterations are reflected in tumor transcriptional heterogeneity. Such mutation‐informed analytical strategies may improve interpretation of genomic alterations beyond mutation frequency alone and provide additional biological context for understanding tumor diversity.

Through multilayer evidence integration, we identified *CDCA8*, *MCM10*, and *TRIP13* as genes consistently associated with *TP53*‐related transcriptional programs. These genes have previously been implicated in cell cycle regulation and genomic stability, biological processes frequently dysregulated in *TP53*‐mutant tumors. *CDCA8* has been associated with chromosome segregation and mitotic progression [38], *MCM10* plays a role in DNA replication initiation [39], and *TRIP13* has been linked to mitotic checkpoint regulation [40]. Because all three genes are also closely related to proliferation, the present data do not establish that they are independent *TP53*‐specific drivers. Rather, the findings support their interpretation as representative components of a *TP53*‐associated proliferative and DNA repair transcriptional state. Experimental studies, including expression‐level validation in HCC cell lines or patient‐derived materials and functional perturbation assays, would be required to determine whether these genes have causal roles in *TP53*‐mutant HCC.

Several limitations of this study should be considered. First, mutation‐associated transcriptional signatures were derived from retrospective public datasets, which may introduce cohort‐specific biases related to etiology, clinical background, sequencing platform, and sample processing. Second, although single‐cell data enabled improved resolution of hepatocyte transcriptional heterogeneity, *TP53* mutation status was not directly measured at single‐cell resolution; therefore, single‐cell results should be interpreted as inferred *TP53*‐associated transcriptional activity rather than direct mutation calls. Third, copy number inference was not incorporated into the final workflow, and the distinction between putative malignant, intermediate, and normal‐like hepatocyte states was based on tissue origin and transcriptional programs rather than direct genomic confirmation. Fourth, the final candidate genes were prioritized computationally and were not validated by qPCR, protein‐level assays, or functional experiments in this study. Fifth, the CellChat analysis provides predicted ligand–receptor communication patterns and does not demonstrate experimentally validated cell–cell interactions. Finally, although external datasets and survival analyses provide supportive evidence, cohort‐specific differences across HCC etiologies and clinical contexts may limit generalizability. Future studies incorporating matched single‐cell genomic and transcriptomic profiling, direct experimental validation, and independent prospective cohorts will be necessary to refine these findings.

Despite these limitations, this study provides a mutation‐informed framework for characterizing transcriptional heterogeneity in HCC. Integrating genomic alterations with transcriptional and cellular phenotypes may improve biological interpretation of somatic mutations and provide additional context for tumor organization. The central takeaway is that *TP53*‐associated transcriptional activity in HCC is linked to proliferative, DNA repair, and stress–response programs and can be localized to specific hepatocyte‐like cellular states through integration of bulk and single‐cell transcriptomic data.

## 5. Conclusion

This study defines a mutation‐informed transcriptional framework associated with *TP53* alteration in HCC through integrative multiomics analysis. These findings provide perspective on tumor transcriptional heterogeneity and contribute to improved interpretation of somatic mutation‐associated transcriptional programs.

## Author Contributions

Sheng Zhang and Guojun Zhang have contributed to the work equally and should be regarded as cofirst authors. Sheng Zhang conducted the computational analyses and prepared the initial draft. Guojun Zhang contributed to data curation and analytical interpretation. Wenxuan Wang and Hong Luo assisted with data organization and manuscript revision. Huaping Chen designed the study, supervised the project, and finalized the manuscript.

## Funding

No funding was received for this manuscript.

## Disclosure

All authors read and approved the final version.

## Ethics Statement

This study was based exclusively on publicly available datasets and did not involve direct collection of human or animal samples by the authors. Therefore, ethical approval and informed consent were not required for this study.

## Conflicts of Interest

The authors declare no conflicts of interest.

## Supporting information


**Supporting Information** Additional supporting information can be found online in the Supporting Information section. Figure S1 provides additional analyses supporting the characterization of TP53 high‐signature hepatocyte populations in hepatocellular carcinoma. It includes pseudobulk comparisons between *TP53* high‐ and low‐score malignant hepatocyte‐like cells, covering principal component analysis, differential expression, representative genes, functional program differences, Hallmark pathway enrichment, and patient‐level program comparisons. It also presents exploratory cell–cell communication analyses, including the definition of tumor‐associated communication groups, aggregate communication strength, significant signaling pathways, and representative ligand–receptor interactions involving *TP53* high‐signature malignant hepatocyte‐like cells (Figure S1). Figure S1: Pseudobulk transcriptional and cell interaction analyses of *TP53* high‐signature hepatocyte populations. (A) Principal component analysis of pseudobulk profiles from *TP53* high‐ and low‐score malignant hepatocytes. (B) Volcano plot showing differential expression between *TP53* high‐ and low‐score malignant hepatocytes. (C) Heatmap of top pseudobulk differentially expressed genes. (D) Representative differentially expressed genes between *TP53* high‐ and low‐score malignant hepatocytes. (E) Comparison of functional transcriptional programs between *TP53* high‐ and low‐score malignant hepatocytes. (F) Hallmark gene set enrichment analysis between *TP53* high‐ and low‐score malignant hepatocytes. (G) Correlation structure between *TP53* signature scores and functional transcriptional programs. (H) Patient‐level comparison of functional transcriptional programs. (I) Communication groups defined across tumor‐associated cell populations. (J) Aggregate communication strength across cell groups. (K) Significant signaling pathways involving *TP53* high‐signature malignant hepatocytes. (L) Top ligand–receptor pairs involving *TP53* high‐signature malignant hepatocytes. (M) Partner‐specific communication strength of *TP53* high‐ and low‐signature malignant hepatocytes.

## Data Availability

The datasets analyzed in this study are publicly available. Somatic mutation, RNA sequencing, and clinical data for TCGA‐LIHC were obtained from The Cancer Genome Atlas (TCGA) via the Genomic Data Commons portal. Single‐cell RNA sequencing data were obtained from the Gene Expression Omnibus (GEO) under Accession Number GSE149614. The external bulk validation dataset GSE14520 was also obtained from GEO. All data used in this study are available from public repositories.
