# Supplementary material for: Somatic Mutation–Informed Transcriptional Architecture of Hepatocellular Carcinoma: A Multiomics Study Centered on TP53
Source: Hum Mutat. 2026 Oct 9;2026:2377057. doi: 10.1155/humu/2377057 (PMC13653071; doi:10.1155/humu/2377057)
Supplement: Supplementary file 1 [file HUMU-2026-2377057-s001.pdf]

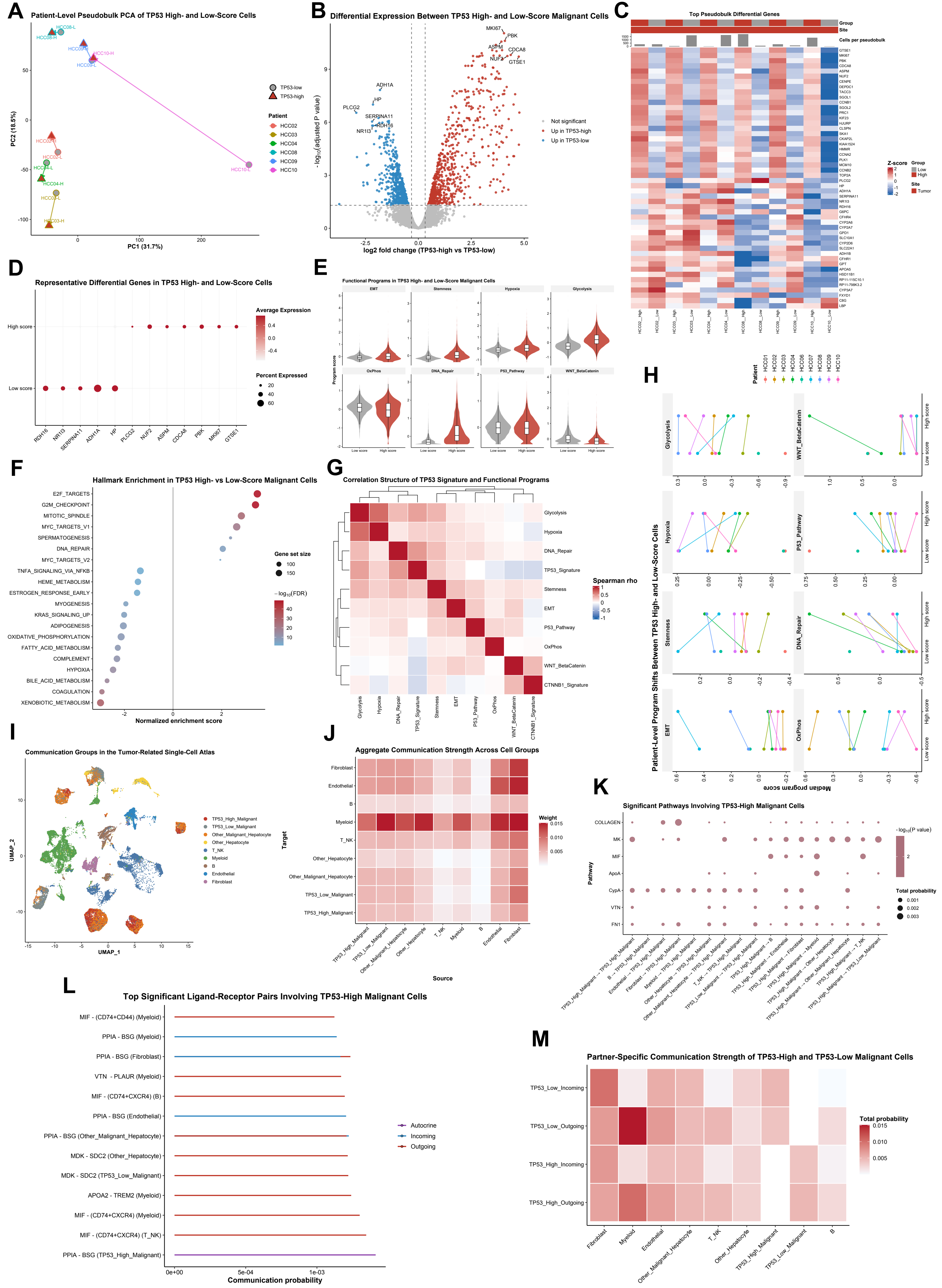

A
Patient-Level Pseudobulk PCA of TP53 High- and Low-Score Cells
PC1 (31.7%)
PC2 (18.5%)
TP53-low
TP53-high
Patient
HCC02
HCC03
HCC04
HCC08
HCC09
HCC10
B
Differential Expression Between TP53 High- and Low-Score Malignant Cells
log2 fold change (TP53-high vs TP53-low)
Not significant
Up in TP53-high
Up in TP53-low
C
Top Pseudobulk Differential Genes
Group
Site
Cells per pseudobulk
Z-score
Low
High
Tumor
D
Representative Differential Genes in TP53 High- and Low-Score Cells
High score
Low score
Average Expression
Percent Expressed
E
Functional Programs in TP53 High- and Low-Score Malignant Cells
EMT
Stemness
Hypoxia
Glycolysis
OxPhos
DNA_Repair
P53_Pathway
WNT_BetaCatenin
Program score
F
Hallmark Enrichment in TP53 High- vs Low-Score Malignant Cells
E2F_TARGETS
G2M_CHECKPOINT
MITOTIC_SPINDLE
MYC_TARGETS_V1
SPERMATOGENESIS
DNA_REPAIR
MYC_TARGETS_V2
TNFA_SIGNALING_VIA_NFKB
HEME_METABOLISM
ESTROGEN_RESPONSE_EARLY
MYOGENESIS
KRAS_SIGNALING_UP
ADIPOGENESIS
OXIDATIVE_PHOSPHORYLATION
FATTY_ACID_METABOLISM
COMPLEMENT
HYPOXIA
BILE_ACID_METABOLISM
COAGULATION
XENOBIOTIC_METABOLISM
Normalized enrichment score
Gene set size
G
Correlation Structure of TP53 Signature and Functional Programs
Glycolysis
Hypoxia
DNA_Repair
TP53_Signature
Stemness
EMT
P53_Pathway
OxPhos
WNT_BetaCatenin
CTNNB1_Signature
Spearman rho
H
Patient-Level Program Shifts Between TP53 High- and Low-Score Cells
Median program score
I
Communication Groups in the Tumor-Related Single-Cell Atlas
TP53_High_Malignant
TP53_Low_Malignant
Other_Malignant_Hepatocyte
Other_Hepatocyte
T_NK
Myeloid
B
Endothelial
Fibroblast
UMAP_1
UMAP_2
J
Aggregate Communication Strength Across Cell Groups
Target
Source
Weight
K
Significant Pathways Involving TP53-High Malignant Cells
COLLAGEN
MK
MIF
ApoA
CypA
VTN
FN1
Pathway
Total probability
L
Top Significant Ligand-Receptor Pairs Involving TP53-High Malignant Cells
MIF - (CD74+CD44) (Myeloid)
PPIA - BSG (Myeloid)
PPIA - BSG (Fibroblast)
VTN - PLAUR (Myeloid)
MIF - (CD74+CXCR4) (B)
PPIA - BSG (Endothelial)
PPIA - BSG (Other_Malignant_Hepatocyte)
MDK - SDC2 (Other_Hepatocyte)
MDK - SDC2 (TP53_Low_Malignant)
APOA2 - TREM2 (Myeloid)
MIF - (CD74+CXCR4) (Myeloid)
MIF - (CD74+CXCR4) (T_NK)
PPIA - BSG (TP53_High_Malignant)
Autocrine
Incoming
Outgoing
Communication probability
M
Partner-Specific Communication Strength of TP53-High and TP53-Low Malignant Cells
TP53_Low_Incoming
TP53_Low_Outgoing
TP53_High_Incoming
TP53_High_Outgoing
Total probability
